# Healthcare resource utilization and economic burden of multiple sclerosis in Chinese patients: results from a real-world survey

**DOI:** 10.1038/s41598-024-64713-1

**Published:** 2024-07-02

**Authors:** Chenhan Sun, Yusheng Jia, Hainan Li, Xuanqi Qiao, Mi Tang, Meiyan Geng, Eddie Jones, James Pike, Mia Unsworth, Min Hu

**Affiliations:** 1 Department of Health Economics, Fudan University School of Public Health, Shanghai, China; 2 Novartis Pharma AG, HEOR & Access Strategy, Value Access, Beijing, China; 3 Adelphi Real World, Central Nervous System, Bollington, UK; 4 Adelphi Real World, Statistics and Data Analytics, Bollington, UK

**Keywords:** Multiple sclerosis, Healthcare resource, Economic burden, China, Real world data, Survey, Demyelinating diseases, Health care economics

## Abstract

Multiple sclerosis (MS) is uncommon in China and the standard of care is underdeveloped, with limited utilization of disease-modifying treatment (DMT). An understanding of real-world disease burden (including direct medical, non-medical, and indirect costs, such as loss of productivity), is currently lacking in this population. To investigate the overall burden of managing patients with MS in China, a cross-sectional survey of physicians and their consulting patients with MS was conducted in 2021. Physicians provided information on healthcare resource utilization (HCRU; consultations, hospitalizations, tests, medication) and associated costs. Patients provided data on changes in their life, productivity, and impairment of daily activities due to MS. Results were stratified by disease severity using generalized linear models, with a p value < 0.05 considered statistically significant. Patients with more severe disease had greater HCRU, including hospitalizations, consultations and tests/scans, and incurred higher direct and indirect costs and productivity loss, compared with those with milder disease. However, the use of DMT was higher in patients with mild disease severity. With the low uptake and limited efficacy of non-DMT drugs, Chinese patients with MS experience a high disease burden and significant unmet needs. Therapeutic interventions could help save downstream costs and lessen societal burden.

## Introduction

Multiple sclerosis (MS) is a chronic, disabling neurological disease. Patients present with multiple symptoms affecting both physical and mental functions, resulting in impaired quality of life for both patients and their caregivers. Studies in Western countries have demonstrated that MS results in considerable direct costs on healthcare systems and indirect costs on society^1,2^. As MS primarily affects younger individuals of working age, the indirect costs of lost productivity are substantial, and have been reported to constitute over one-third of the total costs of MS^3^.

MS is a rare disease in China, with a prevalence rate of 2.44 cases per 100,000 population in 2016, and a higher prevalence in females than males among the estimated 32,000 people diagnosed with MS in that year, which is considerably lower than in other countries with predominantly white populations^4^. A systematic analysis of MS disease burden showed that Chinese patients with MS had almost 70,000 disability-adjusted life-years (DALY) in 2016, reflecting the loss of healthy mobile living years caused by premature death and disability, higher than almost all the other countries analysed^5^.

The standard of MS care in China is relatively underdeveloped, with few treatment choices and limited utilisation of disease-modifying treatment (DMT). Of the five oral DMTs listed for public reimbursement in the Chinese National Reimbursement Drug List (NDRL), only one is classified as high efficacy^6^. A recent study in China reported that the DMT utilization rate for MS patients was only around 30%, and the low accessibility of highly effective DMTs may lead to recurring relapse, disease progression, and enormous expenditure due to poor disease control^7^.

While such data has been thoroughly explored and established in other countries and absolute costs are found to differ between countries, the economic burden of MS in China has not been comprehensively measured in a real-world clinical setting. A national study in China between 2016 and 2018 reported the cost for each hospitalization for MS in China in 2018^8^, while a recent analysis highlighted the increased economic burden of MS in China through the utilization of DMTs^9^. However, neither of these studies considered the overall healthcare resource utilization (HCRU) or the associated costs of managing progressive disease stages in the Chinese setting. In addition, with more drugs gaining access to the Chinese NRDL after 2018, the real-world treatment landscape in China has undergone significant changes since these studies were last conducted.

In view of the unique and evolving treatment landscape in China, which differs from other countries, a comprehensive assessment of the resource utilization and economic burden of Chinese patients with MS in a real-world setting will be of great importance in understanding patients’ unmet needs and assessing the value of emerging treatments. The objective of this study was to describe the overall HCRU and associated costs of managing patients with MS in China, stratified by disease severity.

## Methods

### Study design

This was a cross-sectional survey of physicians (including neurologists and rehabilitation specialists) and their consulting patients, from an analysis of secondary data drawn from the Adelphi multiple sclerosis (MS) disease specific programme (DSP)™ conducted in China in 2021. DSPs are multinational, observational studies collecting information on real-world clinical practice, designed to identify current disease management, and patient- and physician-reported disease impact^10^.

### Participant selection

Physicians were recruited using publicly available lists, and the data collection setting was secondary or tertiary care (public or private hospitals) in nine provinces across China. Physicians were eligible for inclusion provided they were actively involved in making treatment decisions for MS patients and were able to report on their disease history. They were initially contacted via email, with an overview of the study, patient inclusion criteria, a brief description of the questionnaires, and any other logistical aspects of the survey. All physicians gave their consent to taking part in the study after being made aware of their personal data rights.

Participating physicians were invited to complete a pre-specified patient record form questionnaire for six consecutive patients presenting with MS. Neurologists had to be willing to complete a diary of their patients and be consulted by at least three patients with MS per month. The patient record form included detailed questions on patient demographics, MS-related symptoms, HCRU, including hospitalizations, consultations, tests, disease monitoring with clinical assessments, current comorbidities, and treatments.

Patients were eligible for inclusion if they were aged ≥ 18 years, had a physician-confirmed diagnosis of MS, a score on the expanded disability status scale (EDSS)^11,12^ prior to or at the time of data collection (scores range from 0 to 10; patients identified by rehabilitation specialists required a score of 6 or more in order to ensure breadth of severity), and were not involved in a clinical trial. After providing informed consent, each patient with a physician-completed questionnaire was invited to complete a confidential questionnaire that elicited additional information on changes in their life due to MS, including the use of mobility aids and alterations to their home environment. Patients who were in current employment also provided data on their productivity and daily activities using the work productivity and activity index (WPAI)^13^. Patients were encouraged, but not mandated, to complete all questions, so base sizes could fluctuate across different variables. The capture of matched physician and patient forms allowed a linkage between the respondents, and a comparison of clinical and patient outcomes.

### Sample size

As this was primarily a descriptive study, and the sample size was determined by the duration of the survey period, no formal sample size calculations were performed.

### Data collection

#### Healthcare resource utilization

HCRU included the following:

- Hospitalization, length of stay, reason for hospitalization, severity of hospitalization, admission to intensive care unit (ICU);
- Total number of consultations with the consulting physician, and number of consultations with any other healthcare practitioners (HCPs) in the previous 12 months;
- Total number of different types of tests for diagnosis or monitoring of MS in the previous 12 months;
- Any DMT regimens currently prescribed, as well as symptomatic treatment, and concomitant medication currently prescribed.

#### Costs

Variables included direct medical costs, direct non-medical costs, and indirect costs. Direct medical costs were calculated by combining HCRU and unit expenditure extracted from the medical expenditure database in Shanghai^14^, and adjusted to US$ using the average 2021 exchange rate (6.45)^15^. Direct non-medical costs included expenditures as the result of MS, but did not include the direct purchasing of medical services, such as travel, accommodation, and home services (e.g. home modifications). Indirect costs included the lost earnings or productivity by the patient or caregivers related to the morbidity and mortality of illness^16^.

In our study, direct non-medical costs were captured directly from the patient record form and patient self-completion questionnaire, and included the following:

- Frequency of different kinds of equipment, aids, modifications made to adapt for MS;
- Total cost to the patient personally for adaptations due to MS, e.g. purchasing a walking aid, and home infrastructure changes;
- Annual professional caregiver costs.

Indirect costs were calculated based on work productivity reported in the patient self-completion questionnaire, and average income from the China Statistical Workbook^17^ and included:

- Loss of work productivity and income including retirement or long-term sick leave, unemployment due to MS during the previous 12 months;
- Amount of care received from non-professional caregivers and their associated loss of work productivity and income during the previous 12 months.

### Data analysis

Patients were stratified into three groups based on disease severity according to their current EDSS score (mild disease; 0–2.5, moderate disease; 3–5.5, severe disease; 6 +^18,19^).

Categorical variables were summarized by frequencies and proportions. Continuous data were expressed as medians and interquartile ranges (IQR). Demographic characteristics, HRCU, and costs in different groups were compared using Wilcoxon rank-sum test, *χ*^2^ test, or Kruskal–Wallis test. Generalized linear models (GLMs) with log link and gamma distribution^20^ were used to model the association between current EDSS score and costs adjusting for age and sex. All patient record forms were completed online to minimise the possibility of missing data. Missing data within the patient self-completion questionnaire were not explicitly addressed (e.g. imputed) but the base (n) for each variable was reported. Any patients with missing values for a particular variable were excluded from all analyses in which that variable was used. Similarly, the decrease in sample size base across the stratified groups was due to the exclusion of patients whose EDSS status was “not assessed”. All analyses used STATA statistical software version 16.1. A two-tailed p value of less than 0.05 was considered statistically significant.

### Ethical considerations

The DSP complies with all relevant market research guidelines and legal obligations. Data were collected according to European Pharmaceutical Marketing Research Association (EphMRA) guidelines^21^. The study was performed in accordance with relevant guidelines; ethics approval was obtained from Western Institutional Review Board, protocol number: AG8937. The DSP is non-interventional and employs solely retrospective data collection, and no identifiable protected health information was extracted during the study. All participants were voluntarily screened into the Survey and informed of their anonymity and their right to withdraw from participation at any time. All Survey’s adhered to ESOMAR, EphMRA and other relevant national codes of practice.

## Results

### Patient demographics and disease characteristics

A total of 161 physicians (151 neurologists and 10 rehabilitation specialists) provided information on 965 patients. Physicians were evenly distributed across provinces (Beijing Municipality; 12%, Liaoning Province; 9%, Shanghai Municipality; 12%, Jiangsu Province; 17%, Anhui Province; 8%, Hubei Province; 9%, Guangdong Province; 12%, Sichuan Province; 9%, Shaanxi Province; 10%). Physicians saw a mean (standard deviation, SD) of 6.0 (2.77) patients with MS in a typical month, the vast majority of physician time was spent in a public hospital (97.1%, SD 13.75), distributed across primary (17%), secondary (63%) or tertiary (17%) settings.

EDSS scores were available for 370 patients (38%), who formed the patient sample for this analysis. Of these, approximately 36% were categorised with mild disease (EDSS 0–2.5), 35% with moderate disease (EDSS 3–5.5) and 29% with severe disease (EDSS 6 +). Overall, 69% of the patients stratified by EDSS status were female; the median (IQR) age was 40 (34–50) years, evenly divided between those under and those over 40 years old (Table 1).

**Table 1:** Demographic characteristics and disease characteristics of patients overall and stratified by MS severity.

Variable	Overall	Mild disease (EDSS 0–2.5)	Moderate disease (EDSS 3–5.5)	Severe disease (EDSS 6 +)	p-value
Age, years, n (%)	370	133	129	108	0.001
18–30	66 (17.8)	34 (25.6)	18 (14.0)	14 (13.0)	
31–40	131 (35.4)	49 (36.8)	46 (35.7)	36 (33.3)	
41–50	81 (21.9)	34 (25.6)	24 (18.6)	23 (21.3)	
51 +	92 (24.9)	16 (12.0)	41 (31.8)	35 (32.4)	
Median (IQR)	40 (34–50)	37 (30–45)	41 (34–55)	42 (35–56)	< 0.001
Sex, n (%)	370	133	129	108	0.495
Female	254 (68.6)	92 (69.2)	84 (65.1)	78 (72.2)	
Male	116 (31.4)	41 (30.8)	45 (34.9)	30 (27.8)	
Employment status, n (%)	370	133	129	108	< 0.001
Working full time	119 (32.2)	67 (50.4)	38 (29.5)	14 (13.0)	
Working part time	34 (9.2)	10 (7.5)	19 (14.7)	5 (4.6)	
Homemaker	54 (14.6)	19 (14.3)	13 (10.1)	22 (20.4)	
Student	14 ( 3.8)	10 (7.5)	4 (3.1)	0 (0.0)	
Retired	57 (15.4)	11 (8.3)	26 (20.2)	20 (18.5)	
Unemployed	48 (13.0)	12 (9.0)	8 (6.2)	28 (25.9)	
On long term sick leave	44 (11.9)	4 (3.0)	21 (16.3)	19 (17.6)	
Employment status due to MS, n (%)	128	23	47	58	0.129
Long term sick/retired/unemployed	72 (56.3)	9 (39.1)	26 (55.3)	37 (63.8)	
Insurance, n (%)	370	133	129	108	< 0.001
Urban employee basic medical insurance	233 (63.0)	96 (72.2)	86 (66.7)	51 (47.2)	
Urban (and rural) resident basic medical insurance	119 (32.2)	36 (27.1)	38 (29.5)	45 (41.7)	
Other	18 (4.9)	1 (0.8)	5 (3.9)	12 (11.1)	
Patient status, n (%)	370	133	129	108	0.375
Inpatient	95 (25.7)	29 (21.8)	34 (26.4)	32 (29.6)	
Outpatient	275 (74.3)	104 (78.2)	95 (73.6)	76 (70.4)	
Relapses	370	133	129	108	
Relapses in last 12 months, median (IQR)	1 (0–1)	1 (0–1)	1 (0–1)	1 (0–1)	0.054
Duration of most recent relapse (median [IQR]), days	15 (10–30)	14 (7–20)	14 (8–28)	30 (21–61)	< 0.001
Severity of most recent relapse, n (%)	370	133	129	108	< 0.001
Mild	172 (46.5)	108 (81.2)	55 (42.6)	9 (8.3)	
Moderate	157 (42.4)	24 (18.0)	70 (54.3)	63 (58.3)	
Severe	41 (11.1)	1 (0.8)	4 (3.1)	36 (33.3)	

Overall, 41% of patients were in full- or part-time employment. Of the 40% of patients who were on long-term sick leave, unemployed, or retired, this was a direct result of MS in 56% of cases. All patients, either urban employed or rural, were covered by basic medical insurance. In terms of relapsing, 74% of the patients were outpatients, with the most recent relapse lasting a median of 15 (IQR 10–30) days. The duration of relapse was significantly longer in patients with more severe disease (Table 1). Over 50% of the relapses were adjudged to be moderate to severe.

### HCRU and productivity loss

#### Consultations, hospitalizations and tests

Patients had a median of 5 neurologist consultations (IQR 3–7) in the prior 12 months, with as many as 12 visits to an MS specialist, physiotherapist, or rehabilitation specialist, or for traditional Chinese medicine (Table 2). Approximately one third of hospitalizations in the previous 12 months were due to relapses, with significantly more hospitalizations in patients in the higher EDSS group due to worsening symptoms, MS-related accidents, or for rehabilitation (Table 2). Irrespective of EDSS status, patients had a median of 1–2 MRI scans in the previous 12 months, while the number of neurological examinations and CT scans increased significantly with greater disease severity (Table 2).

**Table 2:** HCRU for patients overall and stratified by MS severity.

Variable	Overall	Mild disease (EDSS 0–2.5)	Moderate disease (EDSS 3–5.5)	Severe disease (EDSS 6 +)	p-value
Number of consultations in last 12 months	370	133	129	108	
Neurologist, median (IQR)	5 (3–7)	4 (3–6)	5 (3–6)	6 (4–8)	< 0.001
MS specialist, range	0–6	0–6	0–2	0–1	0.978
Internist, range	0–2	0–2	0–2	0–2	0.076
ER, range	0–3	0–1	0–1	0–3	0.002
Physiotherapist, range	0–12	0–12	0–5	0–5	0.003
Psychiatrist, range	0–4	0–4	0–1	0–1	0.661
TCM, range	0–12	0–12	0	0–2	0.181
Rehabilitation specialist, range	0–12	0	0	0–12	< 0.001
Hospitalizations	370	133	129	108	
Number in last 12 months, median (IQR)	1 (0–2)	0 (0–1)	1 (0–1)	1 (1–2)	< 0.001
ICU, n (%)	3 (0.8)	0 (0.0)	2 (1.6)	1 (0.9)	0.399
Relapse, n (%)	123 (33.2)	41 (30.8)	40 (31.0)	42 (38.9)	0.334
Fall, n (%)	15 ( 4.1)	3 (2.3)	5 (3.9)	7 (6.5)	0.237
Symptoms worsened, n (%)	64 (17.3)	10 (7.5)	16 (12.4)	38 (35.2)	< 0.001
MS related accident, n (%)	16 ( 4.3)	1 (0.8)	3 (2.3)	12 (11.1)	< 0.001
Rehabilitation, n (%)	77 (20.8)	7 (5.3)	26 (20.2)	44 (40.7)	< 0.001
Testing/diagnosis, n (%)	40 (10.8)	15 (11.3)	9 (7.0)	16 (14.8)	0.150
Urinary tract infection, n (%)	4 ( 1.1)	1 (0.8)	1 (0.8)	2 (1.9)	0.687
Tests/scans in last 12 months, median (IQR)	370	133	129	108	
Neurological examination	3 (2–4)	2 (1–3)	3 (2–4)	4 (2–6)	< 0.001
MRI	2 (1–2)	2 (1–2)	2 (1–2)	2 (1–2)	0.061
CT	1 (0–1)	0 (0–1)	1 (0–1)	1 (0–2)	< 0.001

*TCM* traditional Chinese medicine.

#### Medication

Although several DMT options are available for patients with MS in China (Appendix), only 25% of patients in this study were receiving DMTs, with 14% receiving teriflunomide, 4% interferon beta-1b, and 4% fingolimod. Other medications included non-DMTs, such as azathioprine (17%), cyclophosphamide (6%) and rituximab (8%), while some patients received symptomatic treatment, mainly gabapentin (30%), citalopram/escitalopram (29%), or baclofen (27%), Overall, 32% of patients were receiving no drug treatment (Table 3).

**Table 3:** Current treatment for patients overall and stratified by MS severity.

Variable, n (%)	Overall	Mild disease (EDSS 0–2.5)	Moderate disease (EDSS 3–5.5)	Severe disease (EDSS 6 +)	p-value
Disease modifying treatment	370	133	129	108	
Teriflunomide	52 (14.1)	17 (12.8)	24 (18.6)	11 (10.2)	0.155
Interferon beta-1b	15 (4.1)	8 (6.0)	4 (3.1)	3 (2.8)	0.454
Fingolimod	15 (4.1)	8 (6.0)	6 (4.7)	1 (0.9)	0.090
Dimethyl fumarate	5 (1.4)	3 (2.3)	2 (1.6)	0 (0.0)	0.385
Siponimod	8 (2.2)	4 (3.0)	2 (1.6)	2 (1.9)	0.747
Non disease-modifying treatment	370	133	129	108	
Azathioprine	62 (16.8)	9 (6.8)	38 (29.5)	15 (13.9)	< 0.001
Cyclophosphamide	23 (6.2)	5 (3.8)	2 (1.6)	16 (14.8)	< 0.001
Rituximab	28 (7.6)	2 (1.5)	9 (7.0)	17 (15.7)	< 0.001
Tacrolimus	15 (4.1)	2 (1.5)	5 (3.9)	8 (7.4)	0.068
Cyclosporine	18 (4.9)	2 (1.5)	4 (3.1)	12 (11.1)	0.001
Mycophenolate mofetil	9 (2.4)	1 (0.8)	2 (1.6)	6 (5.6)	0.062
Symptomatic treatment	370	133	129	108	
Gabapentin	110 (29.7)	19 (14.3)	40 (31.0)	51 (47.2)	< 0.001
Baclofen	99 (26.8)	28 (21.1)	43 (33.3)	28 (25.9)	0.078
Citalopram	57 (15.4)	14 (10.5)	21 (16.3)	22 (20.4)	0.103
Escitalopram	51 (13.8)	6 (4.5)	21 (16.3)	24 (22.2)	< 0.001
Zolpidem	44 (11.9)	10 (7.5)	17 (13.2)	17 (15.7)	0.125
Pregabalin	39 (10.5)	14 (10.5)	16 (12.4)	9 (8.3)	0.597
Fampridine	28 (7.6)	2 (1.5)	10 (7.8)	16 (14.8)	0.001
Oxybutynin	20 (5.4)	4 (3.0)	2 (1.6)	14 (13.0)	< 0.001
TCM	18 (4.9)	4 (3.0)	7 (5.4)	7 (6.5)	0.430
No drug treatment	117 (31.6)	70 (52.6)	30 (23.3)	17 (15.7)	< 0.001

*TCM* traditional Chinese medicine.

#### Mobility aids

Approximately 39% of patients reported the need for one or more walking aids (cane, walking stick or walking frame) some or all of the time, and 30% had need of a wheelchair, while 62% required support from family or friends. A total of 33% of patients reported making home modifications (Table 4).

**Table 4:** Disease-related resource use and productivity loss in patients overall and stratified by MS severity.

Variable	Overall	Mild disease (EDSS 0–2.5)	Moderate disease (EDSS 3–5.5)	Severe disease (EDSS 6 +)	p-value
Mobility aid, n (%)					
Cane/walking stick, n (%)	370	133	129	108	< 0.001
None of the time	237 (64.1)	101 (75.9)	84 (65.1)	52 (48.1)	
Some of the time	111 (30.0)	29 (21.8)	34 (26.4)	48 (44.4)	
All of the time	22 (5.9)	3 (2.3)	11 (8.5)	8 (7.4)	
Walking frame (e.g. Zimmer frame), n (%)	370	133	129	108	0.001
None of the time	314 (84.9)	125 (94.0)	106 (82.2)	83 (76.9)	
Some of the time	53 (14.3)	8 (6.0)	20 (15.5)	25 (23.1)	
All of the time	3 (0.8)	0 (0.0)	3 (2.3)	0 (0.0)	
Wheelchair, n (%)	369	133	129	107	< 0.001
None of the time	260 (70.5)	114 (85.7)	93 (72.1)	53 (49.5)	
Some of the time	96 (26.0)	17 (12.8)	32 (24.8)	47 (43.9)	
All of the time	13 (3.5)	2 (1.5)	4 (3.1)	7 (6.5)	
Overall frequency of use (e.g. cane/stick/frame), n (%)	370	133	129	108	< 0.001
None of the time	225 (60.8)	99 (74.4)	77 (59.7)	49 (45.4)	
Some/all of the time	145 (39.2)	34 (25.6)	52 (40.3)	59 (54.6)	
Home modification, n (%)	370	133	129	108	
Adapted bathroom	97 (26.2)	18 (13.5)	34 (26.4)	45 (41.7)	< 0.001
Adapted bedroom	56 (15.1)	9 (6.8)	20 (15.5)	27 (25.0)	0.000
Other amendments	36 (9.7)	13 (9.8)	12 (9.3)	11 (10.2)	0.974
Adapted kitchen	7 (1.9)	3 (2.3)	4 (3.1)	0 (0.0)	0.185
None	248 (67.0)	105 (78.9)	85 (65.9)	58 (53.7)	< 0.001
Support from friends and family, n (%)	369	133	129	107	< 0.001
None of the time	142 (38.5)	77 (57.9)	50 (38.8)	15 (14.0)	
Some of the time	203 (55.0)	55 (41.4)	71 (55.0)	77 (72.0)	
All of the time	24 (6.5)	1 (0.8)	8 (6.2)	15 (14.0)	
Productivity loss, median (IQR)	59	5	25	29	
Number of days long term sick/Retired/Unemployed due to MS	1092 (364–2184)	728 (364–1456)	728 (364–1092)	2184 (728–2912)	0.002
Days missed, median (IQR)	113	58	38	17	
Number of days off work in last 12 months due to MS	30 (20–61)	25 (14–35)	61 (30–98)	61 (28–122)	< 0.001
Hours missed, median (IQR)	178	83	67	28	
Hours missed from work in past 7 days	4 (0–12)	2 (0–7)	3 (0–12)	39 (15–45)	< 0.001
Non-professional caregiver weekly hours, mean (SD)	370	133	129	108	
Total hours	40.8 (44.7)	13.2 (27.7)	40.3 (36.5)	75.3 (46.9)	< 0.001

#### Productivity loss

Both patients and caregivers also experienced significant productivity loss, with those patients in current employment reporting taking a median (IQR) of 30 (20–61) days off work in the prior 12 months due to MS, significantly more in patients with higher disease severity (Table 4). Patients required a mean (SD) 41 (45) hours of extra non-professional care per week.

### Costs

**Figure 1:**
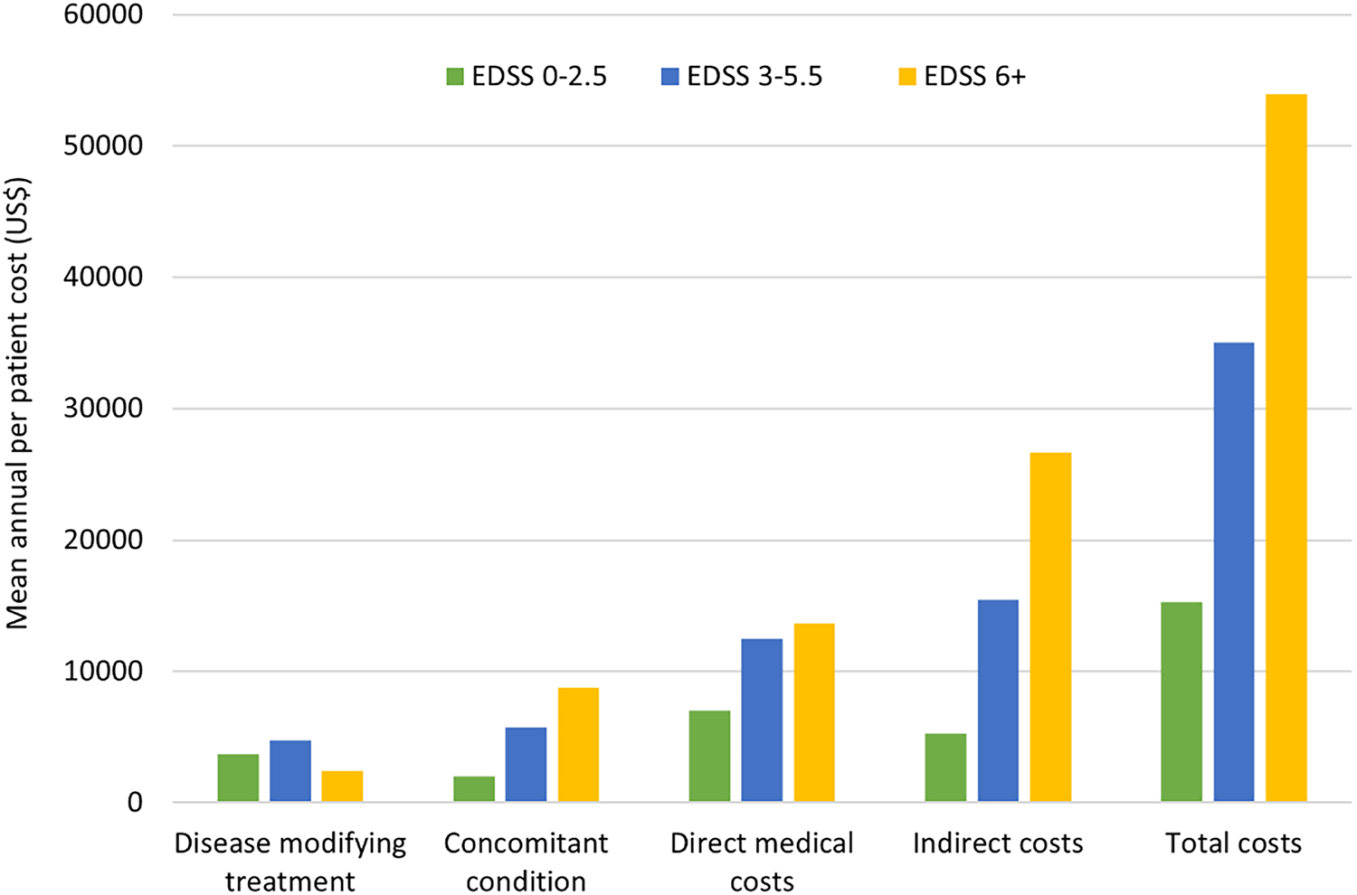
Mean annual per patient costs (US$) related to MS severity.

**Figure 2:**
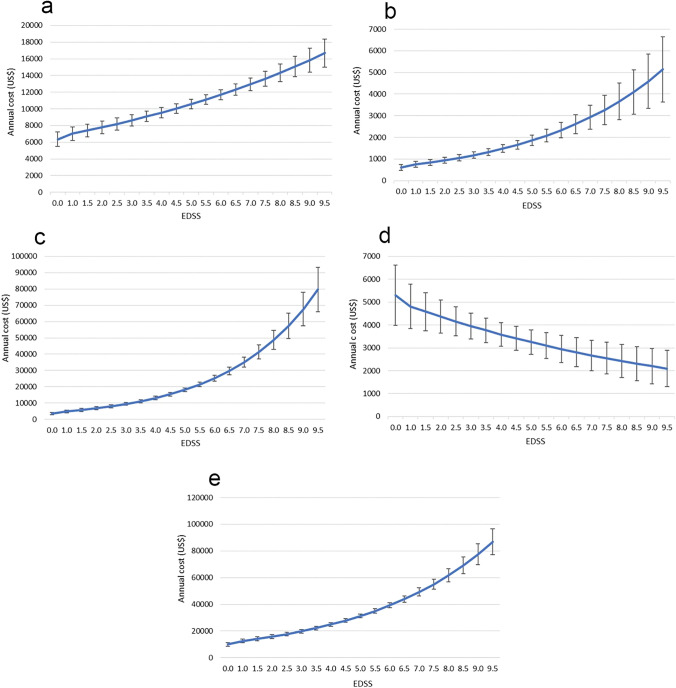
Predicted mean (± SE) total annual direct medical costs per patient by MS severity (**a**); total annual direct non-medical costs per patient by MS severity (**b**); annual indirect costs per patient by MS severity (**c**); annual DMT cost per patient by MS severity (**d**); annual total cost per patient by MS severity (**e**).

The total mean annual cost per patient with MS was CNY215,748 (approximately US$33,450), with costs increasing significantly in line with increasing disease severity (Table 5, Fig. 1). The greatest contributions to the overall costs were use of DMT, treatment of concomitant conditions, and lost productivity for patients and non-professional caregivers (Table 5, Fig. 1). Regression analysis predicted a log-linear increase in direct medical, non-medical, and indirect costs with increasing EDSS score (Fig. 2A–C). Interestingly, DMT use showed a linear decline with increasing disease severity, suggesting more uptake of DMT in the early stages of the disease (Fig. 2D). Total costs were predicted to reach CNY560,000 (approximately $85,000) for patients with the most severe EDSS score (Fig. 2E).

**Table 5:** Mean (± SD) annual costs (CNY) per patient overall and stratified by MS severity.

Costs, mean (SD)	Overall (N = 370)	Mild disease (EDSS 0–2.5) (N = 133)	Moderate disease (EDSS 3–5.5) (N = 129)	Severe disease (EDSS 6 +) (N = 108)	p–value
Direct medical cost					
Hospitalization*	2411 (4441)	1353 (3669)	2608 (4623)	3477 (4823)	< 0.001
Consultation*	330 (228)	314 (290)	307 (189)	378 (174)	< 0.001
Test	3352 (1727)	2604 (1397)	3446 (1703)	4163 (1743)	< 0.001
Symptomatic treatment	6129 (9166)	4106 (7805)	6645 (9150)	8003 (10,271)	< 0.001
Concomitant condition	34,073 (39,072)	12,979 (25,526)	37,052 (38,647)	56,492 (44,038)	< 0.001
DMT	23,824 (50,809)	23,923 (40,890)	30,557 (53,933)	15,660 (56,907)	0.010
Total direct medical costs	70,119 (63,608)	45,278 (51,141)	80,615 (62,418)	88,172 (69,548)	< 0.001
Direct non-medical cost					
Adaptations (e.g. aids, home modifications)	3481 (10,738)	4089 (15,224)	3345 (7248)	2896 (6930)	0.004
Non-medical costs (e.g. transportation)	946 (1401)	1011 (1724)	928 (1452)	1011 (1724)	0.143
Professional care	9293 (41,624)	1454 (9245)	6353 (27,881)	22,456 (68,395)	0.014
Total direct non-medical cost	13,720 (42,899)	6554 (18,154)	10,626 (29,215)	26,241 (68,449)	0.001
Indirect cost**					
Annual patient productivity loss	17,436 (28,604)	8089 (18,628)	20,838 (30,141)	24,882 (33,591)	0.033
Annual non-professional caregiver productivity loss	79,695 (87,277)	25,870 (54,131)	78,744 (71,256)	147,116 (91,597)	< 0.001
Total indirect costs	97,131 (101,688)	33,959 (59,169)	99,582 (84,439)	171,998 (110,393)	< 0.001
Total cost	215,748 (183,258)	98,479 (100,841)	226,001 (142,877)	347,915 (209,759)	< 0.001

*Hospitalization and consultation costs were calculated using unit cost in hospitalization and consultation excluded test, medication, and symptomatic treatment. **Indirect costs were adjusted using GDP CNY 2021.

## Discussion

Our study of patients with MS in China aimed to investigate the impact of disease status on HCRU and associated costs from a real-world perspective. We found that patients with more advanced disease (EDSS 6 +) had greater HCRU, notably more hospitalizations, consultations, and tests/scans than those with milder disease. Patients with the greatest disability, according to EDSS score, incurred the highest costs, especially direct medical costs and indirect costs due to productivity loss of both patients and non-professional caregivers. The cost ratios between different severity levels were consistent with previous reports, at approximately 1 to 2 to 3 for mild, moderate and severe MS in high-income countries^22^, which were comparable to those reported in our study, with ratios of 1 to 2.29 to 3.53, respectively.

The finding in this study about increased HCRU and costs with increased disease severity is reasonable considering the nature of MS disease progression. Our findings indicated that patients with a higher EDSS score were significantly older and had greater disease severity, were less likely to be working, less productive, and required significantly greater care support. Patients with higher EDSS scores also had significantly more hospitalizations, mainly due to worsening symptoms and need for rehabilitation. Not only was there a significant association between EDSS scores and increased disease severity, disease progression, and risk of future progression (p < 0.001), but patients also had significantly more tests/scans in the prior 12 months.

However, despite the association between EDSS score and increasing costs being consistently observed across studies, the total costs per patient differed between studies in different countries. The total mean annual cost per patient with MS in this study was CNY215,748 (approximately US$33,450), which falls within the range of previous studies. A systematic literature review of 29 higher-income countries^22^ estimated the average cost per patient with MS in Europe to be $43,000^23^, while a recent systematic literature review of the costs of MS from 14 studies in low- and middle-income countries (LMICs)^24^ found the total annual direct plus indirect cost per patient to range from $6,247 in Iran^25^ to $58,616 in Argentina^26^. This variation in costs was mainly due to differences in the availability and costs of DMTs, the methodology used to estimate healthcare resource consumption, and the inclusion of informal care and productivity losses.

Our study revealed that the greatest cost drivers were lost productivity for informal caregivers, cost of concomitant conditions, and use of DMT drugs, although their contribution to costs was less in the later disease stage, while the other cost drivers increased, which is aligned with findings in previous systematic literature reviews that drug treatment is the main cost driver in less severe cases of MS, and informal care and productivity losses in the more severe cases^22,24^.

Our findings suggested that higher EDSS scores were also associated with increased need for mobility aids and home modifications, and support from friends and family. As a result, patients with advanced MS, as shown by increased EDSS scores, utilized greater resources, and had higher associated costs, including treatment for concomitant conditions, direct medical costs, and overall productivity loss.

With the relatively low availability and uptake of DMTs in China compared with other countries, Chinese patients are faced with a high disease burden and significant unmet needs^7^. Moreover, the economic burden increases dramatically with disease progression, implying that early control with high-efficacy treatments is critical in improving both the wellbeing and functionality of patients with MS and, at the same time, reducing the economic burden.

Data from real-world studies complement evidence from clinical trials and provides empirical evidence for future studies of the effectiveness of interventions in patients commonly seen in clinical practice in China. Our study has addressed the paucity of real-world data on HCRU and associated costs in China. Moreover, it provides a societal perspective by adding information on the contribution of direct non-medical costs and indirect costs, which have often not been assessed comprehensively in MS. A further strength of the study is the use of GLM to relate EDSS status to outcomes, which illustrated the higher HCRU and costs associated with disease progression.

However, the study has several limitations: firstly, the participant selection is not based on a true random sample of physicians or patients, and the results may not therefore be generalizable to the broader population, since those patients who consult their physician more frequently may be more severely affected than those who do not consult their physician as frequently. Secondly, although physicians were invited to provide data for a consecutive series of patients to avoid selection bias, there were no formal patient selection verification procedures, and participation was influenced by willingness to complete the survey. While recall bias is a common limitation of surveys, the data for these analyses were collected at the time of each patient’s appointment and physicians had access to patient medical records for extraction of historical data, which is expected to reduce this likelihood. Assessment of the target patient group was based on the judgment of the respondent physician and not a formalized diagnostic checklist, but was considered representative of the individual physician’s real-world classification of the patient. Lastly, the cross-sectional design of this study prevents any conclusions about causal relationships.

Increased disease severity in patients with MS in China is associated with a significant rise in HCRU (including consultations and hospitalizations) and the associated direct medical, direct non-medical, and indirect costs. This suggests that interventions that can slow or arrest disability progression could help save downstream costs and lessen societal burden.

### Supplementary Information

Supplementary Information.

## Data Availability

Data collection was undertaken by as part of an independent survey, entitled the Adelphi Real World MS DSP. All data requests for access should be addressed directly to Eddie Jones at Eddie.Jones@adelphigroup.com.
