# Supplementary material for: Healthcare resource utilization and economic burden of multiple sclerosis in Chinese patients: results from a real-world survey
Source: Sci Rep. 2024 Jul 2;14:15183. doi: 10.1038/s41598-024-64713-1 (PMC11219786; doi:10.1038/s41598-024-64713-1)
Supplement: Supplementary file 1 — Supplementary Information. [file 41598_2024_64713_MOESM1_ESM.pdf]

# Healthcare Resource Utilization and Economic Burden of Multiple Sclerosis in Chinese Patients: Results from a Real-World Survey

Jia Y[1†], Sun C[1†], Li H[1], Qiao X[1], Tang M[2], Geng M[2], Jones E[3], Pike J[4], Unsworth M[3], Hu M[1]

[1]Fudan University School of Public Health, Department of Health Economics, Shanghai, China

[2]Novartis Pharma AG, HEOR & Access Strategy, Value Access, Beijing, China

[3]Adelphi Real World, Central Nervous System, Bollington, UK

[4]Adelphi Real World, Statistics and Data Analytics, Bollington, UK

[†]Yusheng Jia and Chenhan Sun contributed equally to this work and share first authorship.

Corresponding author: Dr Min Hu; Email: humin@fudan.edu.cn; Affiliation: Fudan University School of Public Health, Department of Health Economics, Shanghai, China

## SUPPLEMENTAL MATERIAL

### Appendix. Disease modifying treatments licensed for MS in China

| Date of market authorization | Aubagio (teriflunomide) | Gilenya (fingolimod) | Mayzent (siponimod) | Tecfidera (dimethyl fumarate) | Kesimpta (ofatumumab) |
|---|---|---|---|---|---|
| Launch | 2017 | 2019 | 2020 | 2021 | 2021 |
| NRDL Reimbursement | 2019 | 2020 | 2020 | 2022 | 2022 |

NRDL, National Reimbursement Drug List
